# Supplementary material for: Preventing within household transmission of Covid-19: is the provision of accommodation to support self-isolation feasible and acceptable?
Source: BMC Public Health. 2021 Sep 8;21:1641. doi: 10.1186/s12889-021-11666-z (PMC8424161; doi:10.1186/s12889-021-11666-z)
Supplement: Supplementary file 1 — Additional file 1. . [file 12889_2021_11666_MOESM1_ESM.docx]

# Supplementary material 1: Survey to capture views on isolation outside the home

Feedback on a website to help people protect themselves from Coronavirus at home: Can you help?

**
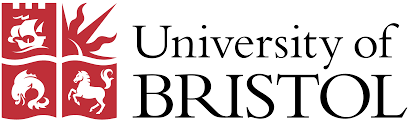
**
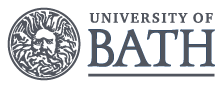

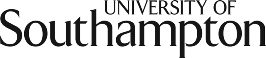


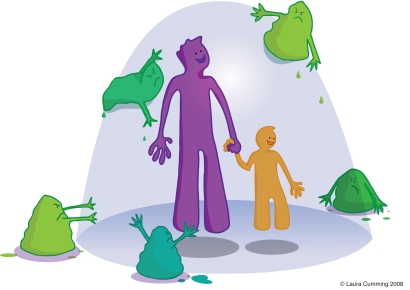


**<*Front page containing full participant information sheet, and second page including consent form>***

In some countries people with coronavirus or people who are at higher risk if they catch coronavirus are offered accommodation to self-isolate outside their own home. This is done so that coronavirus does not spread to other household members, especially if they are at higher risk.

1. How likely would you be to accept an offer of accommodation for you to self-isolate away from your home while you were ill? (for example, in an unused student flat)

[Single choice]

1. I would accept
2. I probably would accept
3. I might accept
4. I definitely would not accept

2. Why?

We'd also like a little bit of information about you. This is so we can make sure the website can help everyone who needs it.

We apologise for asking you to complete these questions again. This survey is anonymous so we can’t link your answers to the information you already gave us on the Germ Defence website.

1. How old are you?

- 18 to 25
- 26 to 40
- 41 to 60
- 61 to 70
- Over 70

1. What is your experience with COVID-19 (the coronavirus)?

- I think I have had the virus
- I think someone I live with has had the virus
- I am at increased risk if I get the virus
- Someone I live with is at increased risk if they get the virus
- None of the above
- Other (Please write more details if possible)

1. If possible, please give us a little more information about your experience with COVID-19.
2. When did you leave full time education? (if you are still in education, please select the stage you are at now)

- Before finishing school
- After finishing school
- After finishing university
- After postgraduate studies

*Other demographic questions may be added as needed to inform current research*

Thank you very much!

We cannot act on all the suggestions we receive, but we will read everything you tell us and use this information to help make Germ Defence as useful as possible.

Please click the submit button below to send your answers.

**Back Submit**
