# Supplementary material for: Preventing within household transmission of Covid-19: is the provision of accommodation to support self-isolation feasible and acceptable?
Source: BMC Public Health. 2021 Sep 8;21:1641. doi: 10.1186/s12889-021-11666-z (PMC8424161; doi:10.1186/s12889-021-11666-z)
Supplement: Supplementary file 2 — Additional file 2. . [file 12889_2021_11666_MOESM2_ESM.docx]

# Supplementary material 2: Semi structured topic guide

**Experiences of risk minimisation practices**

Can you tell me about your experiences of trying to protect yourself and/or other household members during the pandemic?

- What steps did you take?
- Did anything change over time?
- Main challenges/difficulties encountered?
- Day to day living arrangements?
- How do family/household members cope with these practices?
- Other impacts – work, finances, social networks, other care needs?
- Do you have any help during the period of isolation? What kind of help?

**Definitions of isolation and self-isolation**

What does isolation / self-isolation mean to you and your family?

- Is there a similar term/concept in your first language (or language spoken at home)? What is it? Do these terms mean exactly the same thing? What are the differences?
- How do you interpret PHE’s official advice on self-isolation?
- How do you feel about having to self-isolate, or just to isolate in general?
- Do you have family members who need to self-isolate? Do they understand what isolation means and what they need to do?

**Isolation from household members within the home**

*(For those who have been asked to self-isolate within the home)*

Did you or your family members isolate from each other in your home?

- How did you feel about having to isolate at home?
- How did you evaluate the risks of infection (to other family members) and protection? Any specific strategies to enhance protection?
- Challenges encountered with in-home isolation?
- Can you draw a layout or a map of your home during isolation? What were the areas that are risky/contaminated? And what were the safer areas? How did you coordinate day to day lives and movements in relation to perceptions of safety and danger?
- Have you considered living elsewhere for self-isolation? Why or why not?
- What were the biggest concerns you had when you had to do in-home self-isolation?

In some countries, accommodation is offered to help people with coronavirus or people who are at higher risk if they catch coronavirus self-isolate outside their own home. This is done so that coronavirus does not spread to other household members, especially if they are at higher risk.

- Would there be any opportunity within your family or community to self-isolate outside of your home?
- How would you have felt about being offered accommodation for you to self-isolate away from your home either while you were ill, or to protect you while others were ill?
- Prompt – what type of accommodation would you consider?
- What would stop you accepting an offer of this kind?
- What might encourage you to accept an offer of this kind?
- How would you feel about accommodation being offered to high risk members of your household while you were ill?  (Prompts as for 4)
- Is there anything else you would like to say about self-isolation within or outside the home?

**Isolation outside the household**

*(For those who were able to move out of their homes during self-isolation)*

Did you or your family members protect yourselves by moving out of your home?

- How did you feel about having to take these steps?
- How did you evaluate the risks of infection (to other family members) and protection? Any specific strategies to enhance protection?
- Challenges encountered with moving out of the home?
- What were the living arrangements?
- Was there support/care available?

Can you draw a layout or a map of your dwelling during isolation outside your home? What were the areas that are risky/contaminated? And what were the safer areas? How did you coordinate day to day lives and movements in relation to perceptions of safety and danger?

- Have you considered living with your family during self-isolation? Why or why not?
- What were the biggest concerns you had when you had to do self-isolation outside your home?

**Activities during isolation**

We realise that it may not be possible for everyone to stay inside the house all the time. Can you tell me about the times that you did not stay inside the house?

*Prompts*

- *Food supplies & medicine*
- *Work/livelihood*
- *Caring responsibilities*
- *Caring for animals*
- *Outdoor space*

Can you draw a map of where you went when you were outside (e.g. for exercise, food, work)? How far did you go? What were the safe/danger zones? Were there specific routes that you followed?

 Do you feel that restricting your usual activities had an impact on your health and wellbeing in any way?

*Prompts*

- *Were there any aspects that particularly affected you?*
- *Was there anything that affected your physical health?*
- *Was there anything that affected your mental health?*

**Support**

What forms of support did you find you needed to protect yourself and your household?

*Prompts*

- *Practical support*
- *Social support*

What forms of support were you able to get and who from?

*Prompts*

- *Public Health England*
- *Other health services*
- *Your employer/school/university*
- *Friends/family*
- *Neighbours*
- *Voluntary organisations*
- *Do you think there’s any other support that might have been helpful?*

**Information and advice**

What information/advice did you get or find about protecting yourself and/or your household from the virus?

*Prompts*

- *Health services (NHS, PHE, GP)*
- *Government*
- *Internet*
- *Media*
- *Social media*
- *Friends/family*

What did you think of this information or advice?

*Prompts*

- *What sources did you find most reliable?*
- *Was there any difficulty in understanding public health advice?*
- *Have you communicated with anyone if you felt confused about the advice?*
